# Diagnosing Autism Spectrum Disorder Without Expertise: A Pilot Study of 5- to 17-Year-Old Individuals Using Gazefinder

**DOI:** 10.3389/fneur.2020.603085

**Published:** 2021-01-28

**Authors:** Kenji J. Tsuchiya, Shuji Hakoshima, Takeshi Hara, Masaru Ninomiya, Manabu Saito, Toru Fujioka, Hirotaka Kosaka, Yoshiyuki Hirano, Muneaki Matsuo, Mitsuru Kikuchi, Yoshihiro Maegaki, Taeko Harada, Tomoko Nishimura, Taiichi Katayama

**Affiliations:** ^1^Research Center for Child Mental Development, Hamamatsu University School of Medicine, Hamamatsu, Japan; ^2^Department of Child Development, United Graduate School of Child Development, Osaka University, Kanazawa University, Hamamatsu University School of Medicine, Chiba University, and University of Fukui, Suita, Japan; ^3^Healthcare Business Division, Development Center, JVCKENWOOD Corporation, Yokohama, Japan; ^4^Center for Healthcare Information Technology, Tokai National Higher Education and Research System, Gifu, Japan; ^5^Faculty of Engineering, Gifu University, Gifu, Japan; ^6^Department of Neuropsychiatry, Graduate School of Medicine, Hirosaki University, Hirosaki, Japan; ^7^Research Center for Child Mental Development, Graduate School of Medicine, Hirosaki University, Hirosaki, Japan; ^8^Department of Science of Human Development, Faculty of Education, Humanities and Social Sciences, University of Fukui, Fukui, Japan; ^9^Research Center for Child Mental Development, University of Fukui, Fukui, Japan; ^10^Department of Neuropsychiatry, Faculty of Medical Sciences, University of Fukui, Fukui, Japan; ^11^Research Center for Child Mental Development, Chiba University, Chiba, Japan; ^12^Department of Pediatrics, Faculty of Medicine, Saga University, Saga, Japan; ^13^Department of Psychiatry and Neurobiology, Graduate School of Medical Science, Kanazawa University, Kanazawa, Japan; ^14^Research Center for Child Mental Development, Kanazawa University, Kanazawa, Japan; ^15^Tottori University Hospital, Yonago, Japan

**Keywords:** autism spectrum disorder, school-age children, adolescent, Gazefinder, machine learning, Japan

## Abstract

Atypical eye gaze is an established clinical sign in the diagnosis of autism spectrum disorder (ASD). We propose a computerized diagnostic algorithm for ASD, applicable to children and adolescents aged between 5 and 17 years using Gazefinder, a system where a set of devices to capture eye gaze patterns and stimulus movie clips are equipped in a personal computer with a monitor. We enrolled 222 individuals aged 5–17 years at seven research facilities in Japan. Among them, we extracted 39 individuals with ASD without any comorbid neurodevelopmental abnormalities (ASD group), 102 typically developing individuals (TD group), and an independent sample of 24 individuals (the second control group). All participants underwent psychoneurological and diagnostic assessments, including the Autism Diagnostic Observation Schedule, second edition, and an examination with Gazefinder (2 min). To enhance the predictive validity, a best-fit diagnostic algorithm of computationally selected attributes originally extracted from Gazefinder was proposed. The inputs were classified automatically into either ASD or TD groups, based on the attribute values. We cross-validated the algorithm using the leave-one-out method in the ASD and TD groups and tested the predictability in the second control group. The best-fit algorithm showed an area under curve (AUC) of 0.84, and the sensitivity, specificity, and accuracy were 74, 80, and 78%, respectively. The AUC for the cross-validation was 0.74 and that for validation in the second control group was 0.91. We confirmed that the diagnostic performance of the best-fit algorithm is comparable to the diagnostic assessment tools for ASD.

## Introduction

Autism spectrum disorder (ASD) is a heterogeneous neurodevelopmental disorder characterized by atypicality in social communication, and restricted and repetitive behaviors. A recent epidemiological study from Japan reported that the prevalence of ASD is higher than 3% in the general population at the age of 5 years (1). ASD affects the quality of life across the lifespan of the affected individual (2). Various early intervention practices have been developed, some of which are effective and promising (3). Timely intervention is key to better outcomes (4), for which accurate diagnostic assessment is prerequisite.

Regardless of the clinical significance of diagnosis, professionals face challenges inherent to diagnostic assessments of ASD. The first challenge lies in the nature of the diagnosis. As there are no well-established biophysiological diagnostic tests, diagnosis is made solely based on behavioral assessment of children. However, the development of such signs in children is not stable along the time course of diagnosis and can change as they grow. The complexity of social engagement in children generally increases from month to month, especially during a younger age. Furthermore, complex social interactions are affected by the children's cooperativeness, which is further influenced by physical conditions such as hunger and fatigue, as well as their moods and tempers (5–7). Furthermore, no single behavioral sign or trait sufficiently points toward the diagnosis. Therefore, a single diagnostic assessment is usually insufficient to confirm the diagnosis of ASD. To overcome this challenge, standardized tools for diagnosing ASD have been developed, including the Autism Diagnostic Observation Schedule, second edition (ADOS-2) (8) and the Autism Diagnostic Interview-Revised (9), which are widely accepted in research and clinical settings because of their high reliability and validity. However, the use of these tools leads to the second challenge because an interview with these tools followed by the *post-hoc* assessment takes considerable time. Moreover, interviewers need to have the required expertise and undergo training sessions beforehand. Unfortunately, the costs associated with the use of the above methods have restricted the clinical availability of the tools. A recent Australian study reported that only a small proportion of children were assessed using these tools when parents raised concerns over the possibility of their children having ASD, and a “wait-and-see” approach was advised instead (10). This has likely happened in Japan as well, where only 32% of children confirmed to have a diagnosis of ASD at 5 years had had a history of clinical diagnosis of ASD until the fifth birthday (1). Many children with ASD are left undiagnosed and are not provided appropriate interventions even at school age.

Owing to these two challenges that clinicians face in the diagnostic assessment of ASD, models that balance the quality and accuracy of assessment with timeliness and ease are desired (11). To meet this demand, biological/physiological biomarkers have been tested in several studies.

Among them, atypical eye gaze patterns in children with ASD have been tested to determine whether they can serve as candidate markers. Recent advances in eye gaze studies rely heavily not only on advances in technology, but also on the fact that eye gaze patterns reflect both biological and behavioral aspects of ASD (11). Eye gaze patterns are under genetic control (12), and a lack of eye gaze onto human faces measured by eye-tracking devices can be a reflection of lack of eye contact with the examiner, a well-known behavioral diagnostic marker of ASD (13). A recent systematic review pointed out that the effect size resulting from the atypicality of eye gaze patterns in individuals with ASD can have standard deviations (SDs) as large as 0.5 (14, 15). The most consistent finding in these review articles is that individuals with ASD spend looking at the non-social stimuli for longer durations than at the social stimuli, human faces in particular (*social paradigm*); the contrast was independent of age, sex, intelligence quotient (IQ), and other conditions. This is consistent with the social processing theory of ASD (14). This atypical eye gaze pattern was more specifically tested in a preferential viewing paradigm, in which individuals, particularly young children with ASD stare preferentially at geometric figures than at human figures (*preferential paradigm*) (16, 17).

Considering the relative ease of measurement and the biophysiological significance of eye gaze, the atypical eye gaze patterns measured with automated eye-tracking devices can serve as diagnostic markers. In preliminary attempts involving young children, the diagnostic validity and test-retest reliability of eye gaze measurements have been supported (16, 17). However, to the best of our knowledge, no study has tested individuals with ASD in a wide age range. The lack of knowledge is particularly evident among school-aged children and adolescents. Furthermore, previous studies have used eye-tracking devices not specifically developed for individuals with ASD. Since some individuals, especially of young ages, cannot cooperate in keeping their eyes on the monitor, the quickest calibration without any intentional cooperation of the child and minimal duration of stimulus movies should be implemented to validate these attempts in clinical settings. To address this, we have attempted to establish specific eye gaze patterns as a biophysiological markers predicting the diagnosis of ASD, using an eye-tracking system called “Gazefinder” in a broad age range of study participants (18, 19). Novel devices and software were designed, including a calibration movie (5–7 s) and stimulus movies. In the stimulus movies, two paradigms were adopted to test the diagnostic predictability of ASD: the social paradigm and the preferential paradigm. The application of Gazefinder to children does not require any expertise, and it takes <2 min to obtain an output (18, 19). Thus, this system is anticipated to fulfill current demands in ASD diagnosis.

In this study, we propose a computerized diagnostic algorithm for ASD using Gazefinder, implemented with social and preferential paradigms in individuals aged 5–17 years. To realize this, we conducted a multisite study to create a computerized best-fit diagnostic algorithm with satisfactory sensitivity and specificity, and validated it in two ways.

## Methods

### Participants

Two hundred and twenty-two individuals aged between 5 and 17 years were enrolled by physicians at seven research sites and affiliated clinics at Hamamatsu University School of Medicine, Hirosaki University, University of Fukui, Chiba University, Saga University, Kanazawa University, and Tottori University during a 6-month period beginning on 25 February 2018. The seven university clinics are located in small or middle-sized cities and metropolitan areas throughout Japan. All clinics play pivotal roles in providing services for children and adolescents with developmental disabilities in the context of child and adolescent psychiatry and/or pediatric neurology. The reasons for enrolling the participating individuals were as follows: (1) They were previously suspected by psychologists, speech therapists, physicians, or school teachers as having ASD, including “autism” and “Asperger disorder, or (2) they self-nominated to participate in response to the web-based advertisement and have never been suspected to have developmental disorders such as ASD, attention-deficit hyperactivity disorder (ADHD), and learning disabilities. All the participating individuals and their parents were of Japanese ethnicity.

All the legal guardians (i.e., parents in this study) of the participants provided written informed consent, and the participating individuals provided informed assent orally. The study protocol was approved by the ethics committees of the seven research sites and conformed to the tenets of the Declaration of Helsinki.

### Measurement

#### Clinical Evaluation, Screening, and Diagnosis

The initial clinical evaluation by a board-certified psychiatrist or pediatrician included face-to-face behavioral assessment and collection of the developmental history, physical morbidity, and history of medication. This clinical evaluation was followed by screening for ASD using the Pervasive Developmental Disorders Autism Society Japan Rating Scale [PARS, a questionnaire for parents, 57 items (20)], the Strength and Difficulty Questionnaire [SDQ, a questionnaire for parents, 25 items (21)], and the Social Responsiveness Scale in Japanese, second version [SRS-2, a questionnaire for parents, 65 items (22)]. ADHD was screened using the ADHD Rating Scale [ADHD-RS, a questionnaire for parents, 18 items (23)]. General cognitive ability was assessed as indexed by the IQ with the Wechsler Intelligence Scale for Children-fourth edition (WISC-IV) for 215 (97%) individuals, or with the Tanaka-Binet test (Japanese version of the Stanford-Binet Test) for four individuals (2%), or as indexed by developmental quotient (DQ) with the Kyoto Scale of Psychological Development by trained clinical psychologists for three individuals (1%), depending on the participants' mental age. An IQ or DQ lower than 70 was defined as general cognitive delay. An IQ of lower than 70 in WISC-IV is an indication of 2 SD below the population average. The comparability of the IQs derived from the Tanaka-Binet test was tested with the IQ derived from WISC-III, the prior version of WISC-IV (24), and the comparability of the DQs derived from the Kyoto Scale of Psychological Development was tested with the Tanaka-Binet IQ (25).

After the screening tests and assessment of general cognitive abilities, individuals exhibiting positive results to any one of the three screening tests for ASD (PARS, SDQ, SRS-2) were assessed to have a diagnosis of ASD using the ADOS-2 (a semi-structured, play-based observational assessment tool involving interaction with the child and observation of the activities proposed to the child, 11–16 diagnostic algorithm items depending on module) (8), or the Autism Diagnostic Interview-Revised-Japanese Version (ADI-R-JV, a semi-structured interview for parents, 93 items) (9) conducted by trained clinical researchers. In addition, all the participants were assessed to have a diagnosis of ADHD using Conners 3 Japanese version (Conners 3, a questionnaire for parents, 108 items) (26). We did not use both ADOS-2 and ADI-R-JV in our study because of the limited availability of examiners for these tools who had established research reliability with the developers of the instruments. Among 102 individuals with a positive screening result, 81 individuals were assessed only with ADOS-2, nine individuals were assessed only with ADI-R-JV, and 12 individuals were assessed with both ADOS-2 and ADI-R-JV. The remaining 120 individuals were not assessed with ADOS-2 or ADI-R-JV because of negative results in the screening tests.

#### Apparatus

Gazefinder, a system in which a set of devices to capture eye gaze patterns and stimulating movie clips are equipped in a personal computer (PC) with a 19-inch monitor (1280 × 1024 pixels), manufactured by JVC Kenwood Co. Ltd. (Yokohama, Japan), was chosen to measure eye gaze patterns. The technical features of this system have been described in previous studies (18, 19, 27, 28). In brief, corneal reflection techniques enable the device to calculate eye gaze positions on the PC monitor as (X, Y) coordinates in pixel units, at a frequency of 50 Hz (i.e., 3,000 eye gaze positions detected per minute). Using the device, the (X, Y) coordinates provide information regarding where a participating child exactly looked at in the movie clip every 1/50 s when the movie clips were shown to the child on the monitor. The participants were asked to sit in front of the monitor to retain the distance between the face and the monitor at approximately 60 cm. Before the diagnostic measurement of the eye gaze patterns, the calibration of the eye gaze position started automatically and took 5–7 s to complete. The calibration was judged as successful if the child looked at no less than three of the five regions of the calibration movie clip. Otherwise, the calibration movie clip restarted. This movie clip contains a black background with a circular region covered with animated animals, moving from the center to the four corners of the monitor consecutively. After the calibration, 12 short movie clips were automatically presented as experimental stimuli in a fixed order. Between the movie clips, we inserted short attention-grabbing movie clips (2.0 s) to set the eye gaze position at the center of the monitor, prior to the stimuli presentation appearing next. This insertion also canceled out the post-images of the movie clip shown previously. The total time of the movie sequence was 95 s, including time for non-stimulus movie clips.

#### Movie Clips as Stimuli

**Figure 1:**
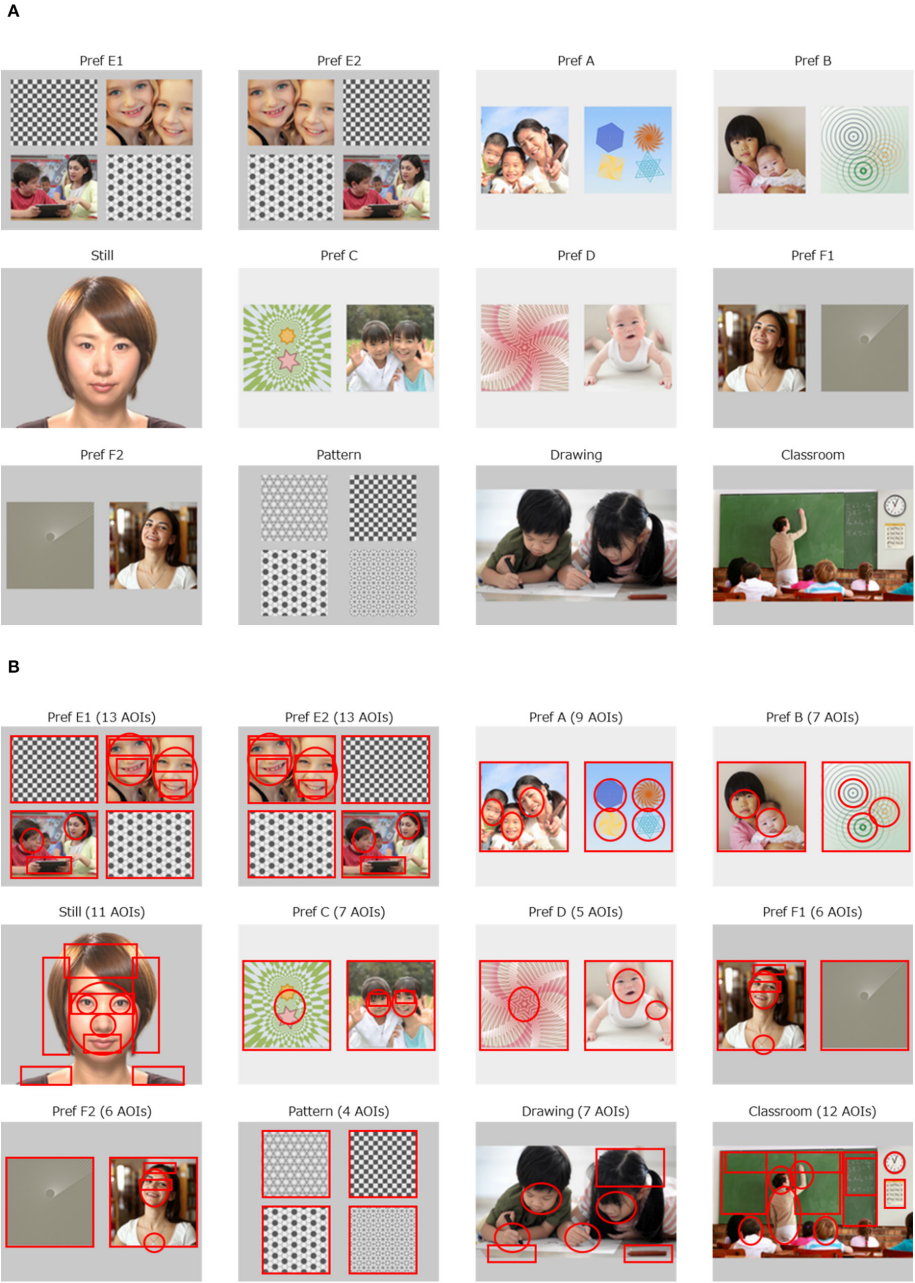
Movie clips implemented with Gazefinder, and the AOIs. **(A)** The 12 movie clips. **(B)** 100 AOIs embedded in 12 movie clips.

Figure 1A presents the 12 movie clips used as stimuli. The social paradigm represented in three movie clips were as follows: a still face of a young amateur actress (“Still,” 4.5 s), two children drawing pictures cooperatively (“Drawing,” 7.0 s), and a teacher teaching a math class in a classroom (“Classroom,” 11.0 s). The preferential paradigm was represented in nine movie clips, in which the visual field on the monitor was divided into two or four regions of equal size, with human figures or objects in parallel. The preferential paradigm movie clips consisted of one movie clip of four graphical patterns (“Pattern,” 7.0 s), and of eight movie clips with human figures with geometric patterns aligned side by side (“Pref A,” 5.0 s, “Pref B,” 4.5 s; “Pref C,” 5.0 s, “Pref D,” 4.5 s, “Pref E1,” 5.0 s, “Pref E2,” 4.5 s, “Pref F1,” 6.0 s, “Pref F2,” 5.5 s). The duration of these movie clips was set identical to that used in our previous studies. The remaining movie clips were kept as short as possible while preserving their context.

**Figure 2:**
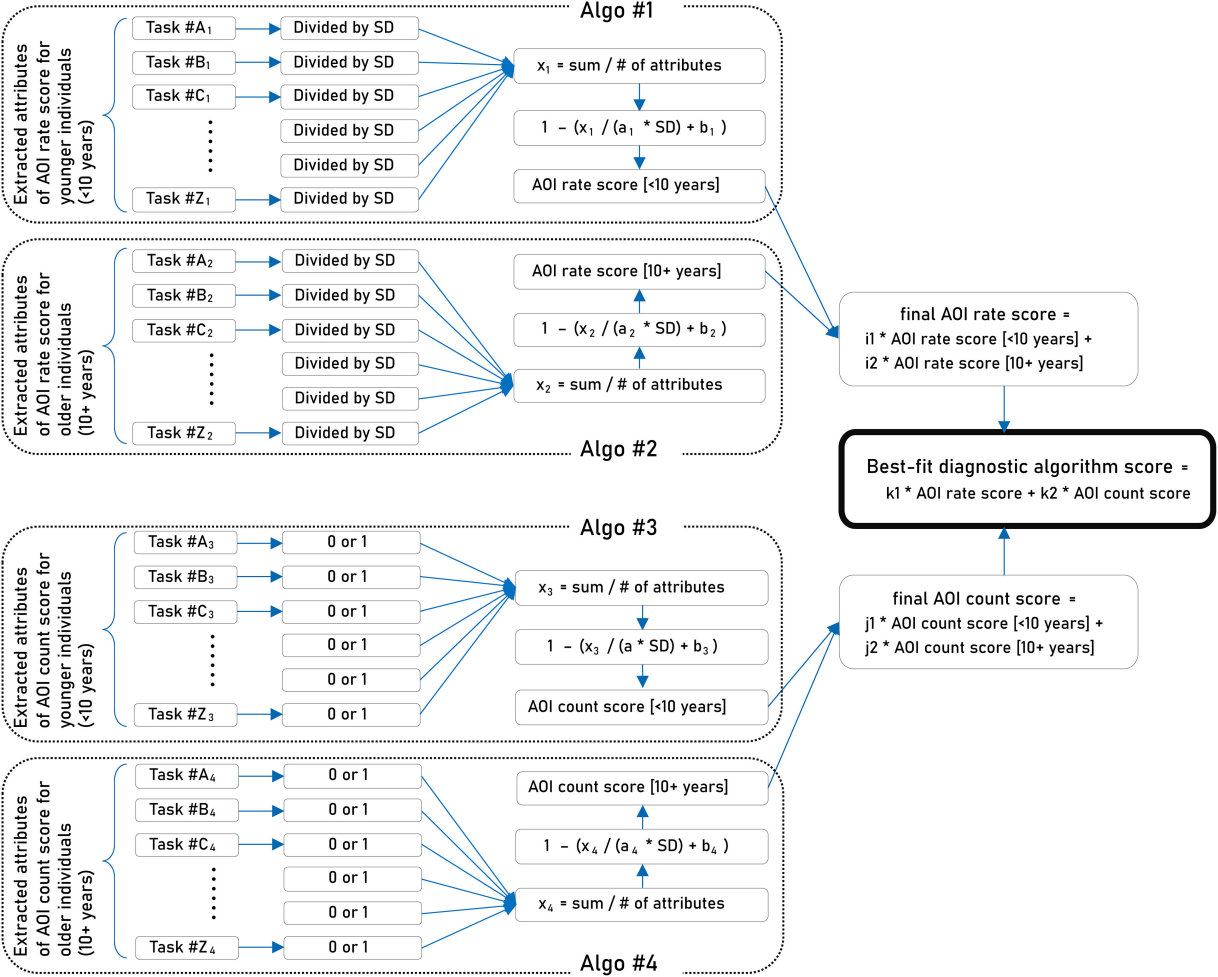
Strategy for creating the best-fit algorithm.

The rationale for creating the movie clips was as follows. As for the social paradigm, there is sufficient evidence to support the fact that a decreased gaze fixation on human faces, especially on eye regions on the monitor is associated with a diagnosis of ASD, regardless of age or IQ (14, 29). We have previously reported that fixation onto the eye region decreases in individuals with ASD at ages of 10 years and older (18). In addition, a decreased amount of gaze fixation onto human figures in social scenes has been reported consistently in individuals with ASD (14). Furthermore, it has been suggested that the quantity of the social content (e.g., the number of human figures presented in the scene) as well as its quality (e.g., human interaction) is related to the amount of reduction in gaze fixation in individuals with ASD (14, 15). Thus, the duration of gaze fixation is measured to contribute to the likelihood of ASD diagnosis, probably in accordance with the number of human figures (2 in “Drawing” and 8 in “Classroom”). Therefore, we presented two children drawing pictures while interacting in the movie clip “Drawing”. As for the preferential paradigm, we have previously confirmed that human figures were more preferentially looked at than non-social figures in typically developing individuals compared to individuals with ASD (18). To control the spatial preference (e.g., adherence to the right half of the visual field) that may be present in some participants with ASD (30), two sets of movie clips were duplicated (“Pref E1” vs. “Pref E2,” “Pref F1” vs. “Pref F2”), but the allocation of the targets (human figures) were exchanged horizontally, and inserted as different movie clips. In the other four sets of movies, the appearance of the side (left or right) of the target (human figures) was balanced.

#### Quantification of Eye Gaze Patterns

Through the sequence of the 12 movie clips, we defined 100 areas of interest (AOIs) in circles or squares on each move clip, specified with x and y axes on the monitor (Figure 1B). We calculated two types of eye gaze indices: the AOI rate score and the AOI count score. The AOI rate score was defined as the percentage of gaze fixation time allocated to each AOI divided by the duration of each movie clip. The AOI rate score was between 0.0 and 1.0 and represents the focus on the object in a dose-response manner (i.e., the higher the AOI rate score, the more intensively the child focused on the object). The AOI count score was a representation of the presence (or absence) of a fixed gaze over each AOI, regardless of the duration of the eye gaze. The possible AOI count score was 0 or 1 and reflects the presence of eye gaze on the AOI, irrespective of possible distractions occurring due to the child focusing on another AOI because of knowledge-driven prediction (31). This is likely to emerge among older individuals. This distraction also occurs when a human agent on the monitor acts as if she/he looked at the individual in front of the monitor (32). As such, we expected that the AOI count score would be more suitable for older individuals. We calculated both the AOI rate scores and AOI count scores separately for all 100 AOIs. In addition, we also calculated two different methods for AOI rate scores applied to the 100 AOIs. The first is to calculate the AOI rate scores of the first 1.0 s and the other is to calculate the AOI rate scores of the first 2.0 s. The intention for this was to generate more attributes with diagnostic value. Specifically, young individuals with ASD have been reported to pay less attention to faces during the initial viewing period (33). As a result, 300 sets of calculation for AOI rate scores and 100 sets of calculation for AOI count scores were applied.

### Analysis

#### Strategy for Creating and Validating the Diagnostic Algorithm

##### Selection of Participants

We selected participants to generate a training dataset to realize the computerized diagnostic algorithm, and an independent dataset for the validation. We first excluded 57 participants from the following analyses because they had a diagnosis of ADHD (*N* = 29), general cognitive delay (*N* = 24), or a clinical diagnosis of epilepsy (*N* = 4). The intention was to minimize the neurophysiological heterogeneity of the subjects included in the dataset, except for the difference between ASD and typical development (TD). The remaining 165 participants were divided into three groups: ASD, TD, and the second control group. The ASD group (*N* = 39) consisted of individuals with a diagnosis of ASD confirmed with ADOS-2 or ADI-R and with a negative screening result for ADHD. The TD group (*N* = 102) consisted of individuals fulfilling negative screening results for both ASD and ADHD. The ASD and TD groups were primarily used as the source of the best-fit computerized diagnostic algorithm and the training and test datasets to check the validity of the best-fit diagnostic algorithm. The second control group consisted of two types of individuals: (1) those with a diagnosis of ASD with a positive screening result for ADHD, and (2) those without a confirmed diagnosis of ASD but with a positive screening result for ASD (the screening result for ADHD can be either positive or negative). The second control group served as an independent sample to validate the diagnostic predictability of the best-fit diagnostic algorithm.

We further divided the ASD and TD groups according to age. Although the social paradigm was assumed to be age-independent (14), the preferential paradigm was reported to distinguish ASD from TD individuals, particularly when the subjects were 10 years and older (18). Considering these, both the ASD and TD groups were divided into younger (<10 years) and older (10 years and older) groups, respectively. We set the age of 10 as the breaking point as in the previous study (18) and because of the statistical reason that 10 was the median age of the individuals in the ASD and TD groups.

##### Extracting Indices (Candidate Attributes)

We calculated the 300 sets of AOI rate scores and 100 sets of AOI count scores for all participating individuals. To extract indices to be included in the best-estimate diagnostic algorithm, the mean values for both AOI rate scores and AOI count scores were compared between the ASD and TD groups for the younger and older age bands. When we found indices that were significantly (*p* < 0.05) associated with the ASD diagnosis or had an effect size (Cohen's *d*) of 0.5 or larger, we retained these as *candidate attributes*, the indices to be included in the next step. To this end, we extracted four sets of candidate attributes based on the AOI rate scores and count scores of the younger and older individuals. To minimize unnecessary weights and to avoid overfitting resulting from choosing multiple AOIs out of one region on a movie clip, we chose only one attribute with the largest effect size out of the candidate attributes that were calculated on the same AOI. This rule also applies to the three AOI rate scores that share the same AOI (the AOI rate scores calculated from the first 1.0 s, from the first 2.0 s, and from the beginning to the end of the movie clip). In addition, to avoid including chance findings with large effect size, we dropped candidate attributes that were extracted from the AOIs with gaze fixation percentage of <20%, which corresponds roughly to a duration of 1.0 s or longer for most movie clips.

##### Creating the Best-Fit Diagnostic Algorithm

We first created four diagnostic algorithms, Algo #1 (the AOI rate scores for the younger individuals), #2 (the AOI rate scores for the older individuals), #3 (the AOI count scores for the younger individuals), and #4 (the AOI count scores for the older individuals), based on the four sets of candidate attributes combined (Figure 2). For each algorithm, the candidate attributes were either divided by the standard deviation, or dichotomized to 0 or 1 and summed, followed by a division by the number of the candidate attributes. These values were fit for a sigmoid function that only took a value between 0 and 1. The next step was to merge Algo #1 and #2 to estimate the final AOI rate score, and to merge Algo #3 and #4 to estimate the final AOI count score. To merge two sigmoid functions, coefficients were estimated automatically to maximize the predictability of ASD diagnosis.

Next, we finalized the best-fit diagnostic algorithm using the two separately estimated algorithm-based scores: the final AOI rate score and the final AOI count score. Before merging the two scores, we chose one algorithm score of a better fit in the younger and older participants separately, to maximize the predictive validity. The fit was assessed with the area under the receiver operating characteristic (ROC) curve (AUC) for each set of comparisons. We then made the selected estimated scores merge smoothly as a continuum along the age range of the participants of 5–17 years (the best-fit algorithm). To merge the two sigmoid functions, coefficients were again estimated automatically to maximize the predictability of the ASD diagnosis. The best-fit algorithm score was made to take values between 0 and 1. In the following analyses, a value of 0.5 or higher of the best-fit algorithm score was assumed as an indicator of the individual under investigation having ASD.

##### Evaluation of the Diagnostic Performance (Cross-Validation)

We evaluated the general classification performance of the best-fit algorithm using the leave-one-out (LOO) method (34). We repeated the procedures described above, including extraction of candidate attributes and formulation of four separate algorithms to be merged into a single best-fit diagnostic algorithm, without the inclusion of one specific individual (LOO algorithm). The removed individual was tested for ASD based on the LOO algorithm. This procedure was iterated for all participating individuals (*N* = 141; cross-validation). We then drew the ROC curves and calculated AUCs for both the best-fit diagnostic algorithm, and votes of the 141 LOO algorithms were used to interpret whether the validity of the best-fit algorithm might have been compromised. To simplify the interpretation, we also calculated the sensitivity, specificity, and accuracy for the best-fit algorithm and for the votes of 141 LOO algorithms separately. The point where the sensitivity and specificity were extracted was set at the Youden J index (i.e., sensitivity + specificity – 1) was maximized (35).

##### Evaluation of the Diagnostic Performance in an Independent Sample

Using the second control group (*N* = 24), we checked the diagnostic validity of the best-fit algorithm independently. Since this group consisted of individuals with ASD with a positive screening result for ADHD (*N* = 17) and individuals with no diagnosis of ASD but with a positive screening result for ASD (*N* = 7), we applied the best-fit algorithm and checked whether the diagnosis predicted with the best-fit algorithm score complied with the real diagnosis using AUC, sensitivity, specificity, and accuracy.

#### Statistics

All statistical analyses were conducted using Stata version 15.1 and R version 3.6.2. To calculate AUC values with 95% confidence intervals, ROC-kit 0.91 was used for resampling. For comparison of two continuous variables, we carried out either the *t*-test or the Kruskal-Wallis test, depending on the distribution. To avoid missing any potential candidate attributes at the early stage of the analyses, we set 0.05 as the significance level.

## Results

### Characteristics of the Participating Individuals

Table 1 shows the demographic and clinical characteristics of the participants. There was no significant difference in the mean age of the participants among the groups. Compared with the TD group, the ASD group showed significantly lower IQ and higher scores on the ASD screening scale (PARS total) and ADHD screening scales (inattention and hyperactivity subscales of ADHD-RS).

**Table 1:** Characteristics of the participants.

	**TD**	**ASD**	**Second control**	Statistics^*^
Number of subjects	102	39	24	
Age in years, mean (SD)	9.5 (4.0)	10.3 (4.0)	10.4 (3.6)	*F*_(2, 162)_ = 0.86, *p* = 0.42
Male sex, number (%)	43 (42%)	30 (77%)	15 (63%)	$\chi_{\left(2\right)}^{2}$=14.6, *p* = 0.001
IQ/DQ, mean (SD)	104.1 (13.6)	94.5 (12.3)	98.7 (14.4)	*F*_(2,162)_ = 7.64, *p* < 0.001 TD>ASD
ASD screening [PARS total], mean (SD)	0.8 (1.1)	7.8 (5.2)	8.0 (7.0)	*F*_(2,162)_ = 70.6, *p* < 0.001 TD < ASD, TD < Second control
ADHD screening [ADHD-RS inattention], mean (SD)	3.3 (3.5)	7.9 (4.8)	11.7 (6.7)	*F*_(2,162)_ = 42.1, *p* < 0.001 TD < ASD, TD < Second control ASD < Second control
ADHD screening [ADHD-RS hyperactivity], mean (SD)	1.9 (3.0)	5.2 (4.4)	7.9 (6.2)	*F*_(2,162)_ = 26.6, *p* < 0.001 TD < ASD, TD < Second control ASD < Second control
Diagnosed as having ASD, number (%)	0 (0%)	39 (100%)	17 (71%)	$\chi_{\left(2\right)}^{2}$= 142.9, *p* < 0.001
Overall gaze fixation percentage, mean (SD)	92.1 (7.3)	89.0 (10.2)	91.2 (8.1)	*F*_(2,162)_ = 2.24, *p* = 0.11

* *Statistically significant results after one-way ANOVA (F tests) were followed by group comparison with Bonferroni correction*.

### Overall Gaze Fixation Percentage (Success of Data Retrieval)

The bottom row of Table 1 shows that the overall gaze fixation percentage values during the measurement using Gazefinder were not statistically different across the groups (92% in the TD group, 89% in the ASD group, 91% in the second control group). The lowest value was 47.4% of a child belonging to the ASD group, but this was the only record below 60%. Out of the 165 participants, 161 showed a value of 70% or higher.

### Extracting Indices (Candidate Attributes)

As for the first steps to create the best-fit diagnostic algorithm, we extracted the four sets of the candidate attributes to be used in the algorithm. The candidate attributes are shown on the corresponding AOIs in association with the AOI rate scores in Supplementary Figures 1, 2 for the younger and older individuals, respectively, and in association with the AOI count scores in Supplementary Figures 3, 4 in the younger and older individuals, respectively.

### Creating the Best-Fit Diagnostic Algorithm

**Figure 3:**
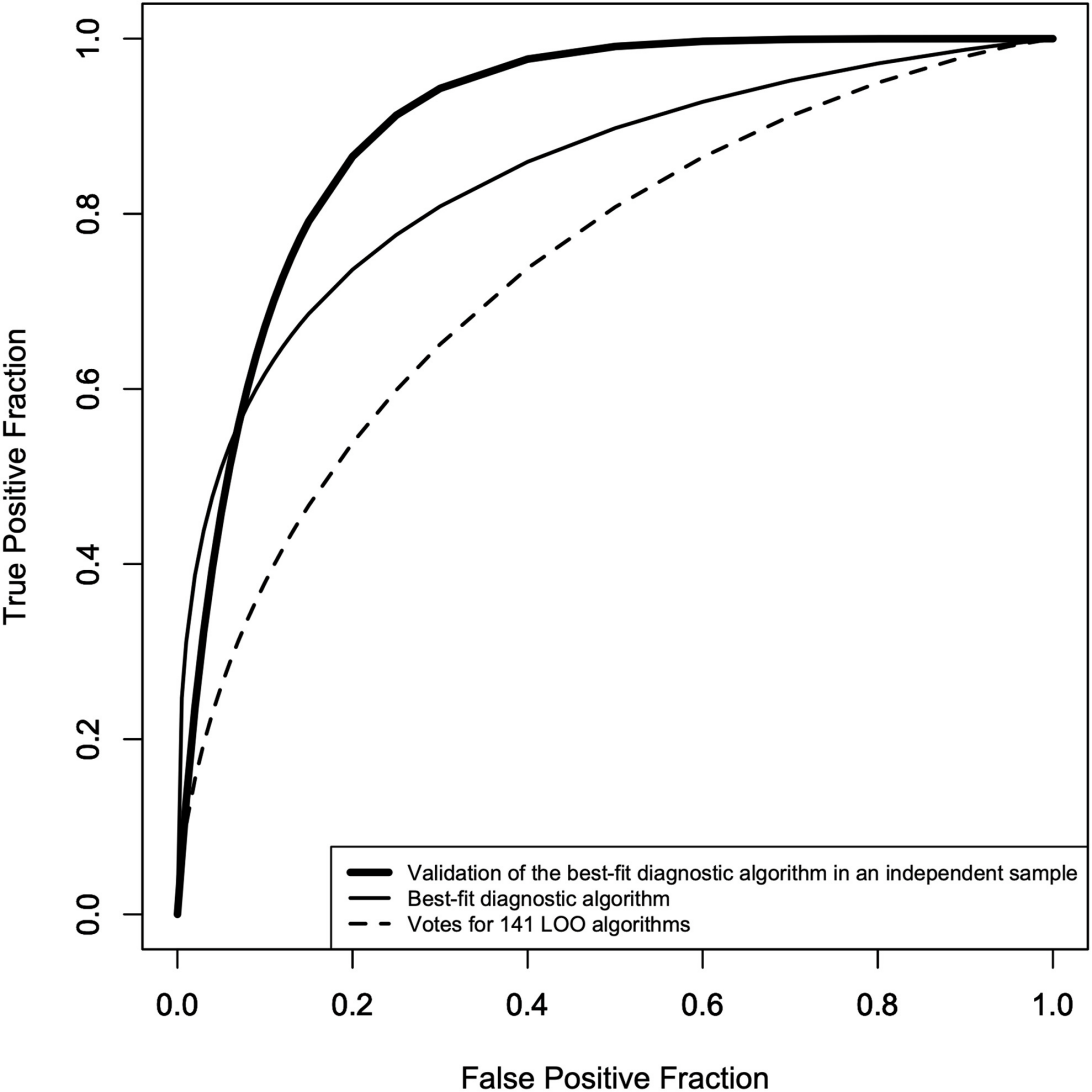
ROC curves of the best-fit diagnostic algorithm* and the votes of the 141 LOO** algorithms. Solid line: ROC curve of the best-fit diagnostic algorithm (AUC = 0.84, sensitivity = 71%, specificity = 80%, accuracy = 78%), dotted line: ROC curve of the votes of the 141 LOO algorithms (AUC = 0.74, sensitivity = 65%, specificity = 70%, accuracy = 67%), thick line: ROC curve of the best-fit diagnostic algorithm in an independent sample (second control group: AUC = 0.91, sensitivity = 87%, specificity = 80%, accuracy = 88%). *The merged algorithm of the final AOI rate score algorithm for age <10 years, and the final AOI count score algorithm for 10 years and over. **Leave-one-out method to cross-validate the best-fit diagnostic algorithm.

Table 2 shows the AUCs for Algo #1, 2, 3, 4, the final AOI rate score algorithms, and the final AOI count score algorithms. To select one algorithm for the two age bands, we found that the final AOI rate score algorithm fit equally for the younger and older individuals (0.82 vs. 0.82) and that the final AOI count score algorithm showed a better fit for the older individuals (0.75 vs. 0.87). For the younger individuals, we selected the final AOI rate score algorithm, and for the older individuals, we selected the final AOI count score algorithm. Merging the two algorithms along the age bands provided the best-fit diagnostic algorithm, of which the ROC curve is shown as a solid line in Figure 3, and the AUC was 0.84, as shown in the first row of Table 3. The sensitivity, specificity, and accuracy were 74, 80, and 78%, respectively. We also checked whether the best-fit diagnostic algorithm showed a good fit for the younger and older participants. The second and third rows of Table 3 show that the AUC, sensitivity, specificity, and accuracy did not differ between the younger and older participants.

**Table 2:** Calculated values of the area under curve (AUC) and their 95% confidence intervals (CIs) for the proposed algorithms for younger and older participants using either AOI rate scores or AOI count scores.

	**Subjects to be tested**	**AUC**	**95% CI**
Algo #1: AOI rate score for the younger individuals	Younger individuals (*n* = 80)	0.83	0.72–0.90
Algo #2: AOI rate score for the older individuals	Older individuals (*n* = 61)	0.83	0.72–0.92
Final AOI rate score algorithm	Younger individuals (*n* = 80)	0.82	0.70–0.90
Final AOI rate score algorithm	Older individuals (*n* = 61)	0.82	0.69–0.92
Algo #3: AOI count score for the younger individuals	Younger individuals (*n* = 80)	0.74	0.63–0.83
Algo #4: AOI count score for the older individuals	Older individuals (*n* = 61)	0.88	0.78–0.95
Final AOI count score algorithm	Younger individuals (*n* = 80)	0.75	0.63–0.85
Final AOI count score algorithm	Older individuals (*n* = 61)	0.87	0.72–0.95

**Table 3:** Diagnostic performance of the best-fit algorithm.

	**Subjects to be tested**	**AUC**	**95%CI**	Sensitivity^**^	Specificity^**^	Accuracy^**^
Best-fit algorithm	All (*n* = 141)	0.84	0.76–0.91	0.74	0.80	0.78
Best-fit algorithm	Younger individuals (*n* = 80)	0.82	0.70–0.90	0.78	0.70	0.76
Best-fit algorithm	Older individuals (*n* = 61)	0.87	0.72–0.96	0.73	0.87	0.75
Votes for 141 LOO algorithms^*^	All (*n* = 141)	0.74	0.64–0.82	0.65	0.70	0.67
Best-fit algorithm	Second control group (*n* = 24)	0.91	0.66–0.99	0.87	0.80	0.88

* *Leave-one-out (LOO) algorithm: an algorithm developed with a procedure identical to that applied to develop the best-fit algorithm without the inclusion of one specific individual (LOO algorithms). The removed individual was tested for the diagnosis of ASD, based on each LOO algorithm. This procedure was iterated for all participating individuals, and the majority of the votes for the 141 LOO algorithm was used as the cross-validated predicted diagnosis (N = 141; cross-validation)*.

** *Sensitivity and specificity were calculated at the point on the ROC where the Youden J index (Sensitivity + Specificity – 1) was maximized*.

### Evaluation of Diagnostic Performance

To show that the high accuracy of the best-fit diagnostic algorithm did not result from overfitting or from chance alone, the diagnostic performance of the best-fit algorithm was first assessed using the LOO method, the result of which is shown as a dotted line in Figure 3 and in the fourth row of Table 3. The AUC was 0.74, which was smaller than the AUC of the best-fit algorithm, and the sensitivity and specificity were 65 and 70%, respectively. We also checked the diagnostic performance of the best-fit diagnostic algorithm in an independent sample (the second control group, *N* = 24). For the 24 participants in this group, we found an AUC of 0.91, with a sensitivity and specificity of 87 and 80%, respectively.

## Discussion

Using Gazefinder, we successfully created the best-fit diagnostic algorithm to discriminate school-aged and adolescent individuals with ASD from typically developing individuals of the same age range, with a sensitivity of 78% and specificity of 80%. The diagnostic performance was tested in two ways: one was a machine-learning procedure called the LOO method and the other was a test in a different, independent sample of the same age range. These two tests of diagnostic performance indicated acceptable to excellent discriminability.

The reported sensitivity and specificity of ADOS-2 (Module 3) were as high as 91 and 66%, respectively, in a large sample of German children with an average age of 10 (36). Similarly, the sensitivity and specificity of ADI-R in a small sample of Japanese children of age 5–9 were 92 and 84%, respectively, and of age 10–19 were 97 and 90%, respectively (9). The diagnostic accuracy of Gazefinder was not better than that of the standard diagnostic tools, but comparable. In addition, the diagnostic validity of Gazefinder was even better than the established screening tools that are available for a wide range of ages. For instance, the Social Communication Questionnaire (37) is a widely used tool available for a wide age range, although the sensitivity and specificity were 64 and 72%, respectively, in a sample of 1–25-year-old individuals (38). There are also a number of screening tools with reported sensitivities and specificities exceeding 80%, although these figures have not been cross-validated or tested in different datasets (39). Considering the applicability of the best-fit algorithm to a wide age range of the subjects, our validated data support the use of the best-fit algorithm in clinical settings as an available alternative to a range of screening tools for detecting ASD, particularly in terms of diagnostic performance.

In addition to the fact that the diagnostic evaluation using Gazefinder was completed within 2 min without any expertise, it is worth noting that a substantial majority of the participating individuals succeeded in completing the examination. Surprisingly, 161 (98%) of the 165 participants kept staring at the monitor for 70% or longer of the total duration of the examination. The four individuals with <70% of the total fixation time were diagnosed as having ASD. Among these four individuals, only two individuals were correctly predicted as having ASD (data not shown in tables). Apparently, the diagnostic accuracy may be decreased if we include individuals with <70% of the overall gaze fixation percentage. One explanation for this observation is that the lower overall gaze fixation percentage by itself may be predictive of ASD. This leads to an understanding that the accuracy of the best-fit algorithm might have been compromised when the overall gaze fixation percentage was lower than the specific cutoff, for example, 70%. Although the overall accuracy of the best-fit diagnostic algorithm was secured, we may set a threshold of 70% as the lowest percentage for securing algorithm-based diagnosis for clinical use, until firm conclusions are drawn.

### Discussion of the Methodology

There was an initial possibility of potential overfitting due to the limited sample size in our results. This has been discussed in the context where the attributes outnumber the observations in machine-learning-assisted neuroimaging studies (40). We have made several attempts to overcome this shortcoming. First, to enhance the efficiency in creating a valid algorithm, we tried to increase the clinical homogeneity among the diagnostic groups. We extracted participants with ASD without any comorbid conditions and TD participants without any suspicions of ASD symptomatology. Second, we avoided building a multi-layered algorithm. Until recently, neural networks, and their applications such as in deep learning, the prominent feature of which is a combination of perceptrons aligned in multiple layers, has been used widely in literature. The major problem inherent to these techniques is overfitting, particularly if the sample size is small, when the network cannot learn itself (41). Therefore, we adopted a single-layered algorithm. Third, we adopted cross-validation using the LOO method (34). Cross-validation is required not only for checking the predictive validity, but also for achieving optimal diagnostic performance (42). LOO is assumed to perform better than other cross-validation methods because the test data are secured not to be used in the training data to form an algorithm. Furthermore, we used a different, independent dataset (the second control group) to be tested with the best-fit diagnostic algorithm. It is worth noting that the independent second control group was a mixture of individuals with ASD with clinical signs of ADHD, and non-TD individuals with subthreshold signs of either ASD or ADHD or both. However, the diagnostic performance of this sample was not compromised. To this end, our validation processes have supported the robust predictive validity of the proposed best-fit diagnostic algorithm.

### Limitations

Despite the fact that the predictive validity of the best-fit diagnostic algorithm was established, potential limitations of the findings should be acknowledged. First, we enrolled a relatively small sample of individuals of Japanese origin. Our stimulus movie was created to be used among non-Japanese people as well, and included actors of various ethnicities. Our findings may be better replicated in a larger sample with different cultural and biological settings. Second, we invented the diagnostic algorithm based on responsivity to social stimuli; however, this is only one aspect of the broad behavioral spectrum of ASD. Particularly, we have not established that our measure reflects the symptoms of restricted interest and repetitive behaviors (RRBs). Furthermore, the predicted diagnosis does not indicate the severity of the symptoms, as the diagnostic algorithm has been designed to monitor whether responsivity to specific stimuli was observed or not. Thus, Gazefinder has immense possibility for customization in the future. Third, we did not investigate whether the indices we collected were associated with clinical correlates, severity, or prognosis. In a previous study using eye-tracking devices, children with ASD who were more oriented toward social images were shown to have better language and higher IQ scores (16). The clinical applicability of Gazefinder can be further developed in this direction in the context of treatment monitoring. Fourth, we did not assess social anxiety symptoms. In a previous study, gaze avoidance was reported in adolescents with either social anxiety disorder or ASD, but delayed orientation to the eye regions was observed in the latter. We did not examine whether the reduced gaze fixation to the eye regions results from delayed orientation or from orienting in a direction outside the eye regions; this should be addressed in the future. Fifth, we did not assess the participants of our study with both ADOS-2 and ADI-R-JV; we assessed them with only one of these tools. Among 39 individuals with ASD in the analysis, five were assessed only with ADI-R, and two individuals among these were over 10 years. This may compromise the diagnostic accuracy because of higher likelihood of recall biases, although the number of such individuals is minimal. Sixth, we excluded individuals with ADHD and general cognitive delay. Although this exclusion will allow the algorithm to be more sensitive to ASD, the general clinical applicability of the diagnostic algorithm may be limited in clinical settings, where individuals with ASD are frequently comorbid with ADHD and/or cognitive delay. In our future study, we may include individuals with or without ADHD and compare them with individuals with ASD using the diagnostic algorithm. Finally, since we did not conduct full diagnostic assessments for screen-negative individuals, we might have overlooked ASD diagnoses in this group of individuals. However, this is unlikely since our thorough clinical assessments were conducted by trained psychiatrists or pediatricians, all of whom have an experience of joining clinical/research workshop of ADOS-G or ADOS-2 and some have established research reliability with the developers, followed by the consistent negative results for all the three screening tests for ASD.

### Clinical Applicability

In typical community settings, individuals with ASD are expected to be diagnosed at certain stages during childhood (43). However, more than half of the children, adolescents, and young adults with confirmed diagnosis of ASD do not have a history of clinical diagnoses related to ASD, as was reported in a community survey in the last century from the US (44). This was reported a decade ago, although the situation appears to remain the same at present. A more recent study from Japan pointed out that only 32% of children confirmed to have ASD at 5 years of age had any history of neuropsychiatric/neuropediatric diagnosis until their fifth birthdays, meaning that more than half of the children with ASD are left undiagnosed at 5 years of age or even older (1). This may be due to the lack of appropriate chances to be screened, although general health checkups during childhood are a rule in most developed countries. The challenges inherent to the diagnostic evaluation of ASD, particularly in the community setting, should be resolved with ease and without expertise. We propose the computerized best-fit diagnostic algorithm implemented in Gazefinder as a solution to this.

Despite the diagnostic accuracy and convenience of the diagnostic algorithm, standard diagnostic procedures in clinical settings, such as ADOS-2, should not be replaced with diagnosis with Gazefinder. We assume that a diagnostic evaluation with Gazefinder is a mechanical one and thus should be followed with an expert clinical diagnosis. Presently, clinical evaluation may not be readily available in countries where trained manpower is limited, and mechanical diagnosis alone can result in a false sense of security among caregivers. However, in such countries, we can propound that Gazefinder can function as a screener and thereby reduce the burden on experts including pediatricians, child psychologists, and child psychiatrists. We should minimize the drawbacks and maximize the advantages of using Gazefinder in the future. In order not to give false sense of security when a false negative result is provided, the cutoff point should be adjusted to increase the sensitivity.

We have confirmed that the diagnostic performance of the best-fit algorithm is comparable to standard diagnostic tools and is even better than current screening tools for ASD. Diagnostic evaluation using Gazefinder is secured in more than 90% of the participants and adolescents. Thus, the proposed best-fit diagnostic algorithm is ready to be used in clinical settings and to be tested in clinical trials. We have drafted and submitted the protocol to the Japanese supervisory authorities, and currently, a clinical trial is under way. Clinical trials using Gazefinder to establish diagnostic validity in countries other than Japan are also appreciated.

## Data Availability Statement

The datasets presented in this article are not readily available because sharing the data with the third parties not listed in the original protocol has not been approved by the Ethical Committees. Requests to access the datasets should be directed to Kenji J. Tsuchiya, tsuchiya@hama-med.ac.jp.

## Ethics Statement

The studies involving human participants were reviewed and approved by the Ethics Committee of Hamamatsu University School of Medicine and Hospital, the Ethics Committee of University of Fukui, the Ethics Committee of Hirosaki University School of Medicine and Hospital, the Ethics Committee of Graduate School of Medicine and School of Medicine, Chiba University, the Ethics Committee of Saga Medical School Faculty of Medicine, Saga University, the Ethics Committee of Kanazawa University School of Medicine, and the Ethics Committee of Tottori University Faculty of Medicine. Written informed consent to participate in this study was provided by the participants' legal guardian/next of kin. Written informed consent was obtained from the individual(s), and minor(s)' legal guardian/next of kin, for the publication of any potentially identifiable images or data included in this article.

## Author Contributions

KT, SH, THara, MN, MS, HK, YH, MM, MK, YM, and TK: study concept and design. KT, MS, TF, HK, YH, MM, MK, YM, and THarada: resources arrangement, clinical evaluation, and measurement. KT, SH, THara, MN, and TN: analysis and interpretation of data. KT, THara, and TN: drafting of manuscript. KT and TK: obtained funding. All authors contributed to critical revision of manuscript for important intellectual content and approved the submitted version.

## Conflict of Interest

SH and MN are employed by JVCKENWOOD Corporation. The remaining authors declare that the research was conducted in the absence of any commercial or financial relationships that could be construed as a potential conflict of interest.

## Supplementary Material

Supplementary Figure 1 Candidate attributes^*^ of AOI rate scores^**^ among the individuals of younger than 10 years. AOIs outlined with red lines represent parameters with a positive effect (i.e., AOI rate score was higher in individuals with ASD) and AOIs outlines with blue lines represent parameters with a negative effect (i.e., AOI rate score was lower in individuals with ASD). ^*^Attributes that were significantly (*p* < 0.05) associated with the diagnosis of ASD or had an effect size of Cohen's d with 0.5 or larger. ^**^Percentage of fixation time allocated to the AOI divided by duration of each movie clip.

Supplementary Figure 2 Candidate attributes^*^ of AOI rate scores^**^ among the individuals of 10 years and over. AOIs outlined with red lines represent parameters with a positive effect (i.e., AOI rate score was higher in individuals with ASD) and AOIs outlines with blue lines represent parameters with a negative effect (i.e., AOI rate score was lower in individuals with ASD). ^*^Attributes that were significantly (*p* < 0.05) associated with the diagnosis of ASD or had an effect size of Cohen's d with 0.5 or larger. ^**^Percentage of fixation time allocated to the AOI divided by duration of each movie clip.

Supplementary Figure 3 Candidate attributes^*^ of AOI count scores^**^ among the individuals of younger than 10 years. AOIs outlined with red lines represent parameters with a positive effect (i.e., AOI count score was higher in individuals with ASD) and AOIs outlines with blue lines represent parameters with a negative effect (i.e., AOI count score was lower in individuals with ASD). ^*^Attributes that were significantly (*p* < 0.05) associated with the diagnosis of ASD or had an effect size of Cohen's d with 0.5 or larger. ^**^Presence (or absence) of countinous fixed gaze ofer the AOI; takes the value of either 0 or 1.

Supplementary Figure 4 Candidate attributes^*^ of AOI count scores^**^ among the individuals of 10 years and over. AOIs outlined with red lines represent parameters with a positive effect (i.e., AOI count score was higher in individuals with ASD) and AOIs outlines with blue lines represent parameters with a negative effect (i.e., AOI count score was lower in individuals with ASD). ^*^Attributes that were significantly (*p* < 0.05) associated with the diagnosis of ASD or had an effect size of Cohen's d with 0.5 or larger. ^**^Presence (or absence) of countinous fixed gaze ofer the AOI; takes the value of either 0 or 1.
