# Supplementary figures and images for: Diagnosing Autism Spectrum Disorder Without Expertise: A Pilot Study of 5- to 17-Year-Old Individuals Using Gazefinder
Source: Front Neurol. 2021 Jan 28;11:603085. doi: 10.3389/fneur.2020.603085 (PMC7876254; doi:10.3389/fneur.2020.603085)

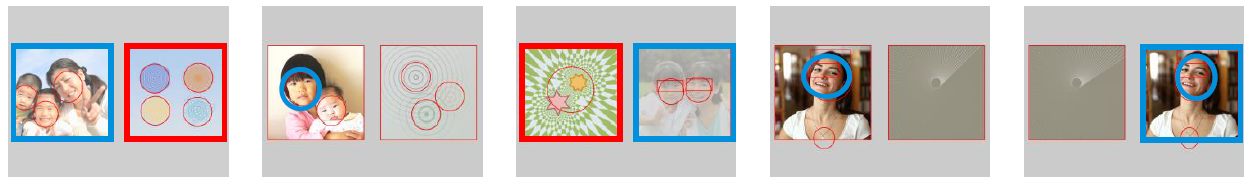

Supplement: Supplementary Figure 1 — Candidate attributes* of AOI rate scores** among the individuals of younger than 10 years. AOIs outlined with red lines represent parameters with a positive effect (i.e., AOI rate score was higher in individuals with ASD) and AOIs outlines with blue lines represent parameters with a negative effect (i.e., AOI rate score was lower in individuals with ASD). *Attributes that were significantly (p < 0.05) associated with the diagnosis of ASD or had an effect size of Cohen's d with 0.5 or larger. **Percentage of fixation time allocated to the AOI divided by duration of each movie clip. [file Image_1.TIF]

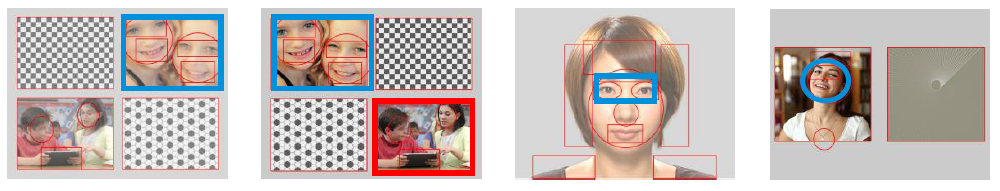

Supplement: Supplementary Figure 2 — Candidate attributes* of AOI rate scores** among the individuals of 10 years and over. AOIs outlined with red lines represent parameters with a positive effect (i.e., AOI rate score was higher in individuals with ASD) and AOIs outlines with blue lines represent parameters with a negative effect (i.e., AOI rate score was lower in individuals with ASD). *Attributes that were significantly (p < 0.05) associated with the diagnosis of ASD or had an effect size of Cohen's d with 0.5 or larger. **Percentage of fixation time allocated to the AOI divided by duration of each movie clip. [file Image_2.TIF]

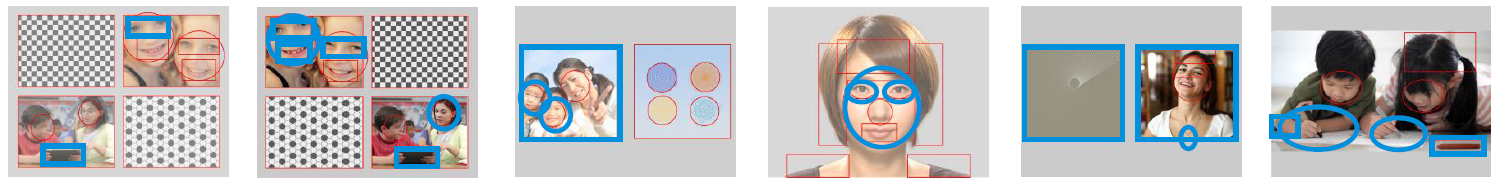

Supplement: Supplementary Figure 3 — Candidate attributes* of AOI count scores** among the individuals of younger than 10 years. AOIs outlined with red lines represent parameters with a positive effect (i.e., AOI count score was higher in individuals with ASD) and AOIs outlines with blue lines represent parameters with a negative effect (i.e., AOI count score was lower in individuals with ASD). *Attributes that were significantly (p < 0.05) associated with the diagnosis of ASD or had an effect size of Cohen's d with 0.5 or larger. **Presence (or absence) of countinous fixed gaze ofer the AOI; takes the value of either 0 or 1. [file Image_3.TIF]

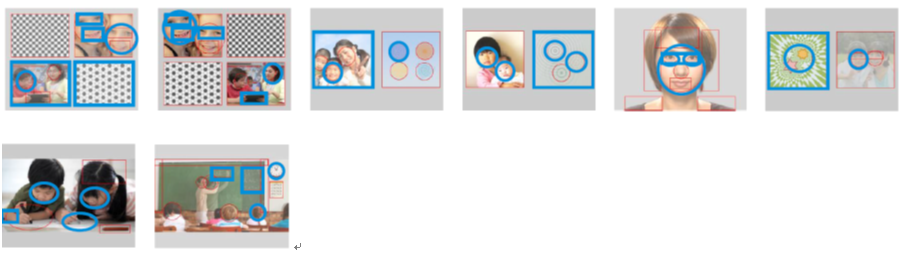

Supplement: Supplementary Figure 4 — Candidate attributes* of AOI count scores** among the individuals of 10 years and over. AOIs outlined with red lines represent parameters with a positive effect (i.e., AOI count score was higher in individuals with ASD) and AOIs outlines with blue lines represent parameters with a negative effect (i.e., AOI count score was lower in individuals with ASD). *Attributes that were significantly (p < 0.05) associated with the diagnosis of ASD or had an effect size of Cohen's d with 0.5 or larger. **Presence (or absence) of countinous fixed gaze ofer the AOI; takes the value of either 0 or 1. [file Image_4.TIF]
